# Characterization of CTX-M-15-Klebsiella pneumoniae from inpatients and outpatients of a teaching hospital

**DOI:** 10.12688/f1000research.53221.1

**Published:** 2021-06-04

**Authors:** Muzaheed, Naveed Sattar Shaikh, Saeed Sattar Shaikh, Sadananda Acharya, Shajiya Sarwar Moosa, Mohammad Habeeb Shaikh, Faisal M. Alzahrani, Amer Ibrahim Alomar

**Affiliations:** 1 Department of Clinical Laboratory Science, College of Applied Medical Sciences, Imam Abdulrahman Bin Faisal University, Dammam, Saudi Arabia; 2 Department of Medicine, People’s University of Medical and Health Sciences, Nawabshah, Pakistan; 3 Department of Public Health, College of Public Health, Imam Abdulrahman Bin Faisal University, Dammam, Saudi Arabia; 4 Department of Anatomy, College of Medicine, Imam Abdulrahman Bin Faisal University, Dammam, Saudi Arabia; 5 College of Medicine, Imam Abdulrahman Bin Faisal University, Dammam, Saudi Arabia

**Keywords:** K. Pneumoniae, ESBL, TEM, SHV, CTX-M-15

## Abstract

Background

The presence of Extended-spectrum β-lactamase (ESBL) positive bacteria in hospital setting is an aggravating influential factor for hospitalized patients, and its consequences may be hazardous. Therefore, there is a need for rapid detection methods for newly emerging drug-resistant bacteria. This study was aimed at the molecular characterization of ESBL-positive *Klebsiella pneumoniae* isolates recovered from clinical samples.

Methods

A total of 513 *K. pneumoniae* isolates were obtained from various clinical samples during June 2019 to May 2020. The collected isolates were investigated for antimicrobial susceptibility (antibiogram), and PCR and DNA sequencing were performed to analyse the ESBL genes.

Results

Among the 513 isolates, as many as 359 (69.9%) were ESBL producers and 87.5% were multi-drug resistant, while none had resistance to imipenem. PCR scored 3% blaTEM, 3% blaSHV, and 60% blaCTX-M-15 genes for the tested isolates.

Conclusion

The study showed that CTX-M-15 was the major prevalent ESBL type among the isolates. Additionally, all the isolates were susceptible to carbapenems. Screening and detection of ESBL tests are necessary among all isolates from the enterobacteriaceae family in routine microbiology laboratory to prevent associated nosocomial infections. A larger study is essential to understand molecular epidemiology of ESBL producing organisms to minimize morbidities due to these multidrug resistant organisms.

## Introduction

The emergence of Extended-spectrum β-lactamase (ESBLs) producing organisms has imposed a great threat on majority of the antibiotic classes, particularly cephalosporins. ^1^ The situation worsens when the patient infected with ESBL-producing organism is administered an antibiotic to which the organism is resistant. ^1^ Clinical practitioners globally have been facing problems related to plasmid mediated ESBL producing *Klebsiella pneumoniae* ( *K. pneumoinae*). These plasmids contain genes encoding resistance to antibiotics such as aminoglycosides, sulfonamides, tetracycline, chloramphenicol, and quinolones. ^2^ Cephalosporins are β-lactam antibiotics that are widely used in clinical practice and in the treatment of bacterial infections. ^3^ In addition, *K. pneumoniae* is reported in nosocomial infections and community acquired infections. ^4^

A study conducted by Maltezou et al. ^5^ revealed that the incidence rate of ESBL producing *K. pneumoniae* infection was 16.71% for Northern Europe, 24.41% for Southern Europe, 58.71% for Eastern Europe, 28.2% for the Asia Pacific region, 51.9% for South America, and 12.3% for North America. ^5^

CTX-M-type β-lactamase, has become more predominant than conventional SHV and TEM-type ESBL, which have covered an extensive range of clinically significant bacteria and over a wide environmental area. ^6^ Moreover, strains that yield ESBL often reveal resistance to antibiotics belonging to other classes (i.e. aminoglycosides, quinolones, and sulfonamides); making its management more complicated. ^7^

Consequently, this class of bacteria makes a need to ensure the reporting of ESBL producers in clinical isolates and detection of newly emerging drug resistant isolates. The changing trends of drug resistant ESBL-producing *K. pneumoniae* need time to watch for management, confinement and isolation of patients in hospitals before discharge. It is also necessary to monitor the prevalence and types of ESBLs and define the appropriate therapeutic options accordingly. Hence, the purpose of the current study was to observe the current trends of ESBL producing *K. pneumoniae* drug resistant patterns and to identify the occurrence of ESBL genes in Sindh region of Pakistan.

## Methods

### Isolation and identification

**Figure 1.**
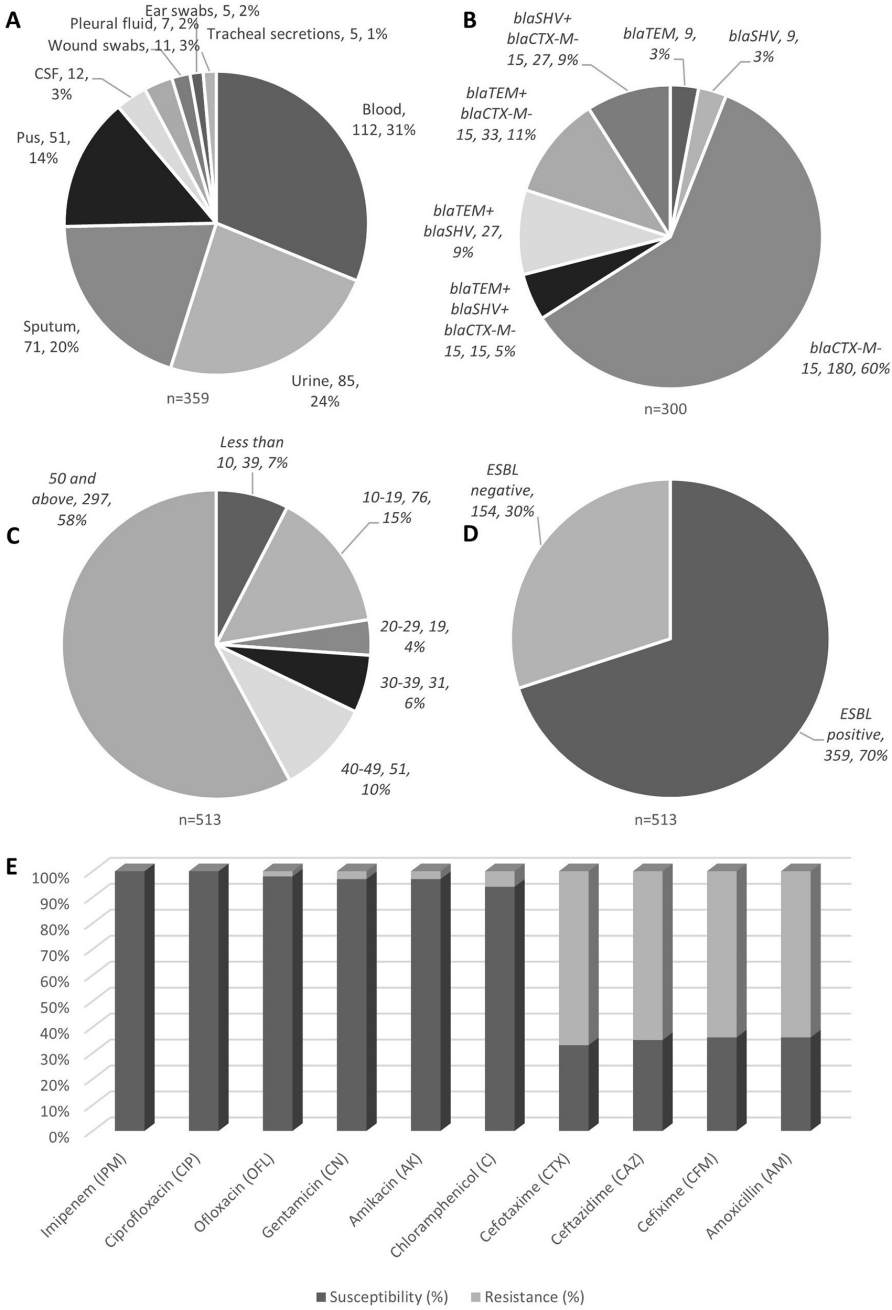
*K. pneumoniae* isoaltes obtained from various clinical samples showed high prevalence as blood borne infections (A); genotypic distribution showed *bla*CTX-M-15 to be highly prevalent (B); age group wise analysis showed elderly group of 50 years old and above showed high incidences (C); ESBL positivitiy to be 70% among all isolates tested (D), with their antibiotic susceptibility profile (E).

A total of 1458 non-duplicate clinical samples were collected between June 2019 and May 2020 from outpatient and inpatient wards of People's University of Medical and Health Sciences, Nawabshah, Pakistan through its diagnostics and research laboratory. The samples were not specifically collected for this research, they were used after the completion of routine laboratory investigations upon the permission of the concerned laboratory's authority. The isolation of bacteria was carried out by conventional bacteriological techniques and identification was confirmed by API 20E identification kit (BioMerieux, France). ^8^ As many as 513 *K. pneumoniae* isolates from various clinical samples and sites ( Figure 1A) were obtained and included in the present study.

### Inclusion and exclusion criteria

Patients referred by their primary physicians for screening tests (such as blood complete count and erythrocyte sedimentation rate (ESR)) were recruited in the study. The clinical samples including blood, sputum, urine, cerebrospinal fluid, tracheal secretions, and pus samples were collected by standard techniques ( Figure 1A). The clinical samples, which yielded the *K. pneumoniae* were selected for further assessment, while others were excluded. The demographic data was retrieved from clinical survey that included age, gender, specimen type, town, and susceptibility patterns ( Table 2).

### Antimicrobial susceptibility test

Antimicrobial susceptibility testing (AST) was performed by Kirby Bauer method on Mueller-Hinton agar plates (Beckton Dickinson, Sparks, MD, USA) according to Clinical Laboratory Standards Institute (CLSI). ^8^ Disks (Beckton Dickinson) comprising of the following antibiotics were used: cefixime 30 μg, amoxicillin 30 μg, ceftazidime 30 μg, cefotaxime 30 μg, Ofloxacin 10 μg, imipenem 10 μg, amikacin 30 μg, gentamicin 10 μg, ciprofloxacin 5 μg and chloramphenicol 30 μg.

### ESBL screening and confirmation by phenotypic methods

Double disk diffusion methods were used to detect ESBL using four antibiotic disks: cefotaxime 30 μg, cefotaxime with clavulanic acid 10 μg, ceftazidime 30 μg, and ceftazidime with clavulanic acid 10 μg. A more than 5 mm increase in zone of inhibition width for whichever antimicrobial agent tested in combination with clavulanic acid against its zone of inhibition when tested alone was labelled as ESBL positive. ^9^ The inoculum and incubation conditions were the same as recommended for standard disk diffusion. *K. pneumoniae* ATCC 700603 (American type culture collection, ATCC, Manassas, USA) was used as quality control strain.

### Detection of ESBL genotypes by PCR amplification

Bacterial genomic DNA was seperated by boiling the young bacterial colony in 50μl distilled water in a hot bath set at 98°C. This DNA template was used for all PCR reactions. Amplification of *bla*TEM, *bla*SHV and *bla*CTX-M-15 ESBL genes was done on GeneAmp PCR System 9700 (Applied Biosystems, Foster City, CA, USA). The primers used are given in Table 1. The PCR yields were run on 1% agarose gels and observed in UV light. Purified PCR products of CTX-M genes were sequenced on an Applied Biosystems 3730 DNA analyzer (Applied Biosystems, Foster City, CA, USA).

**Table 1.** List of primers used.

ESBL Gene	Primer	Sequence (5′-3′) *	Product size (bp)	Annealing temperature (°C)
*bla*_TEM_	Forward	ATGAGTATTCAACATTTCCGTG	861	55
	Reverse	TTACCAATGCTTAATCAGTGAG		
*bla _SHV_*	Forward	ATTTGTCGCTTCTTTACTCGC	1051	60
	Reverse	TTTATGGCGTTACCTTTGACC		
*bla*_CTX-M-15_	Forward	CACACGTGGAATTTAGGGACT	996	50
	Reverse	GCCGTCTAAGGCGATÁAACA		

* Ref: Muzaheed *et al.,* 2008 (32).

### Data analysis

The data were analyzed by Statistical Package of Social Sciences (SPSS) version 21.0. Continuous and categorical variables were analyzed by *t*-test and Chi-square testing, respectively (these tests can also be carried out on the open source software R). Variance of resistance levels of various antimicrobials in ESBL producing isolates versus non-ESBL producing isolates were calculated by the Fisher exact test. The data was analyzed at 95% Confidence interval (p ≤ 0.05 indicates a significant difference).

## Results

During the study period, a total of 513 (35.18%) *K. pneumoniae* were isolated. From those 513 positive cultures, 411 (80.1%) belonged to males and 102 (19.8%) to females (p = 0.0001). The demographic characteristics of patients including age, gender, rural or urban, and clinical interventions are summarized in Table 2. The results show that the majority of patients were in the older age group (50 and above) and lived in urban areas. The categories of patient's ages have been shown in Figure 1C. The mean age of the total study subjects was 48.5 ± 8.9 years.

**Table 2.** Demographic characteristics and interventions in study population (n = 513).

Specimen	*Klebsiella pneumoniae*	
	N	%
Age (mean ± SD)	47 ± 17.5 (range: 9-56 years)	
Male	411	80.1
Female	102	19.8
Rural	124	24.17
Urban	389	75.8
Children	115	22.41
Old age	398	77.58
History of interventions Nasogastric intubation Intravenous line Central venous line Urinary catheters Tracheotomy Endotracheal intubation Surgery	76 319 11 217 9 23 19	14.81 62.18 2.14 42.3 1.75 4.39 3.7

Out of 513 isolates 359 (69.9%) were ESBL producing *K. pneumoniae* ( Figure 1D). The incidences of ESBL was higher in males as compared to females. The frequency of ESBL producing *K. pneumonia*e from different samples (n = 359) has been shown in Table 3 and Figure 1A. The means ± SD age of patients with ESBL positive and negative was 47 ± 17.5 years and 47.5 ± 12.8 years, respectively (p = 0.093).

**Table 3.** Frequency of ESBL - *K. pneumoniae* in various clinical specimens (n = 513).

Specimen	ESBL Positive *Klebsiella pneumoniae*	
	N	%
Blood	112	21.8
Sputum	71	13.8
Pus	51	9.9
Cerebrospinal fluid	12	2.3
Ear swabs	5	0.97
Wound swabs	11	2.14
Pleural fluid	7	1.36
Urine	85	16.5
Tracheal secretions	5	0.97
Total	359/513	69.9

The results show that 64% of *K. pneumoniae* in our sample were resistant to Amoxicillin and Cefixime. In addition, an important drop of 30–40% was perceived in the susceptibility for all Cephalosporins. However, *K. pneumoniae* presented a dissimilar sensitivity rate to Chloramphenicol, Ofloxacin, Gentamicin and Amikacin with 94%, 98%, 97%, and 97%, respectively and depicted in Table 4.

**Table 4.** Antibiotic resistance pattern.

Name of antibiotics	Susceptibility (%)	Resistance (%)
Ceftazidime (CAZ)	35	65
Cefotaxime (CTX)	33	67
Cefixime (CFM)	36	64
Amoxicillin (AM)	36	64
Ofloxacin (OFL)	98	02
Imipenem (IPM)	100	00
Ciprofloxacin (CIP)	100	00
Chloramphenicol (C)	94	06
Gentamicin (CN)	97	03
Amikacin (AK)	97	03

Regarding the PCR and DNA sequencing of ESBL genotypes, it was found that all of the ESBL-positive *K. pneumoniae* isolates had one or more ESBL genes that were tested in the present study and presented in Table 5. Overall, 83.56% (300/359) of *K. pneumoniae* isolates were positive for one or more ESBL genes. The PCR assay and DNA sequencing results indicated the following frequencies of ESBL genotypes: 85% *bla*CTX-M-15 gene, 26% *bla*SHV gene, and 28% *bla*TEM gene.

**Table 5.** Prevalence of ESBL genotypes.

Genotypes	Incidences n (%) *
*bla*_TEM_	9 (3%)
*bla _SHV_*	9 (3%)
*bla*_CTX-M-15_	180 (60%)
*bla*_TEM+_ *bla*_SHV *+*_ *bla*_CTX-M-15_	15 (5%)
*bla*_TEM+_ *bla*_SHV_	27 (9%)
*bla*_TEM+_ *bla*_CTX-M-15_	33 (11%)
*bla*_SHV *+*_ *bla*_CTX-M-15_	27 (9%)

* Overall Total CTX-M-15 is 255 [85]; SHV = 78 [26] and TEM = 84 [28].

## Discussion

ESBL's are the major causes of β-lactam antibiotic resistance, particularly in *K. pneumoniae*, which is amongst the most common gram-negative bacteria belonging to *Enterobacteriaceae* families. ^10^ A study from Ziauddin University Karachi, Pakistan has showed that ESBL producing *K. pneumoniae* frequency is 84.16% amongst reported cases. ^11^ Similarly, Saleem et al. (Aga Khan University, Karachi, Pakistan) have reported a frequency of 80% of ESBL producing *K. pneumoniae*. ^12^ These findings were higher as compared to the results presented in the current study, which showed that the frequency of ESBL producing *K. pneumonia* is 69.6% with a higher incidence in male than in female patients. In this repect, the frequency of EBSL producing *K. pneumoniae* varies from one institution/hospital to another. Several factors could govern this variation such as; the use of antibiotics, drug dose, infection control measures, treating physicians, and the duration of drug therapy - all of these elements may cause a difference in results. The frequency of ESBL producing *K. pneumoniae* in previous research conducted at Aga Khan University Karachi, Pakistan was 30.1% and 47.8%, which is low compared to the present study, as well as, previous studies conducted by Mansouri et al. ^10^ and Ejaz et al. ^13^ A Pakistani study found a frequency of ESBLs producing *K. pneumoniae* of 70%, ^14^ which is in agreement with our study.

Carbapenems are often the final influential therapy of choice to treat infections resulting from MDR *Enterobacteriaceae.* Based on our study and other studies, 100% sensitivity was seen with Imipenem, which have been found to be the most effective antibiotics on the isolates that produce ESBLs. This is an important result of the present study because many infections can be treated with Carbapenems. ^12, 15^

*bla*CTX−M is a predominant genotype in this area of the subcontinent. A further study from Pakistan has reported that 72% of ESBL producing strains had the *bla*CTXM−15 gene, which was higher than the prevalence of *bla*CTX-M gene reported in the present study. ^16^ Limited studies from different regions have also shown a higher prevalence of the *bla*CTX-M genotype amongst strains including 84.7% (Chile), whereas a very high prevalence of 98.8% was reported in China and, significantly, a low prevalence of 13.6% in Tanzania. ^17– 19^

In conclusion, the findings of the current study emphasizes the urgent need for screening and surveillance of ESBL producing *K. pneumoniae* in routine microbiology laboratories for early detection, prompt intervention, and successful clinical management before exacerbation of the infection magnitude. In this regard, the current findings may be a valuable contribution to the medical literature providing physicians with an updated prevelance status of ESBL producing *K. pneumoniae.* Finally, the limited sample size along with the limited time frame are two common limitations, which could affect the outcomes of the study.

## Data Availability

All data underlying the results are available as part of the article and no additional source data are required.

## References

[1] Ali Abdel Rahim KA Ali Mohamed AM : Prevalence of Extended Spectrum β-lactamase-Producing Klebsiella pneumoniae in Clinical Isolates. *Jundishapur J Microbiol.* 2014;7(11):e17114-e. 10.5812/jjm.17114 25774279 PMC4332241

[2] Dallenne C Da Costa A Decre D : Development of a set of multiplex PCR assays for the detection of genes encoding important beta-lactamases in Enterobacteriaceae. *J Antimicrob Chemother.* 2010;65(3):490–5. 10.1093/jac/dkp498 20071363

[3] Peer Maroof Ahmad MAT Bashir AF Ahmed K : Extended Spectrum-β-Lactamase producing Klebsiella pneumoniae at a tertiary care setup in Kashmir, India: Comparative phenotypic detection and antimicrobial susceptibility pattern. *Rev Infect.* 2010;1(2):124–33.

[4] Munoz-Price LS Poirel L Bonomo RA : Clinical epidemiology of the global expansion of Klebsiella pneumoniae carbapenemases. *Lancet Infect Dis.* 2013;13(9):785–96. 10.1016/S1473-3099(13)70190-7 23969216 PMC4673667

[5] Maltezou HC : Metallo-beta-lactamases in Gram-negative bacteria: introducing the era of pan-resistance? *Int J Antimicrob Agents.* 2009;33(5):405e1-7. 10.1016/j.ijantimicag.2008.09.003 19095416

[6] Pishtiwan AH Khadija KM : Prevalence of blatem, blashv, and blactx-M Genes among ESBL-Producing Klebsiella pneumoniae and Escherichia coli Isolated from Thalassemia Patients in Erbil, Iraq. *Mediterr J Hematol Infect Dis.* 2019;11(1):e2019041-e. 10.4084/MJHID.2019.041 31308917 PMC6613628

[7] Mahon CR Lehman DC Manuselis G : *Textbook of Diagnostic Microbiology - E-Book: Elsevier Health Sciences.* 2014.

[8] Matuschek E Brown DF Kahlmeter G : Development of the EUCAST disk diffusion antimicrobial susceptibility testing method and its implementation in routine microbiology laboratories. *Clin Microbiol Infect.* 2014;20(4):O255–66. 10.1111/1469-0691.12373 24131428

[9] Muzaheed DY Adams-Haduch JM Shivannavar CT : Faecal carriage of CTX-M-15-producing Klebsiella pneumoniae in patients with acute gastroenteritis. *Indian J Med Res.* 2009;129(5):599–602. 19675391

[10] Mansouri S Kalantar Neyestanaki D Shokoohi M : Characterization of ampc, CTX-M and mbls types of β-lactamases in clinical isolates of Klebsiella pneumoniae and Escherichia coli producing Extended Spectrum β-lactamases in Kerman, Iran. *Jundishapur J Microbiol.* 2014;7(2):e8756-e. 10.5812/jjm.8756 25147671 PMC4138687

[11] Afridi FI Farooqi BJ Hussain A : Frequency of extended spectrum beta lactamase producing enterobacteriaceae among urinary pathogen isolates. *J Coll Physicians Surg Pak.* 2011;21(12):741–4. 22166694

[12] Saleem AF Qamar FN Shahzad H : Trends in antibiotic susceptibility and incidence of late-onset Klebsiella pneumoniae neonatal sepsis over a six-year period in a neonatal intensive care unit in Karachi, Pakistan. *Int J Infect Dis.* 2013;17(11):e961–5. 10.1016/j.ijid.2013.04.007 23759260

[13] Ejaz H Ul-Haq I Mahmood S : Detection of extended-spectrum β-lactamases in Klebsiella pneumoniae: comparison of phenotypic characterization methods. *Pak J Med Sci.* 2013;29(3):768–72. 10.12669/pjms.293.3576 24353625 PMC3809290

[14] Bari F Shah H Wazir R : Frequency and detection of extended spectrum betalactamase in Escherichia coli and Klebsiella pneumoniae: A study at lady reading hospital peshawar. *J Postgrad Med Inst.* 2015;29(4).

[15] Sourav chakraborty KM Sarker PK Zahangir Alam MD : Prevalence, antibiotic susceptibility profiles and ESBL production in Klebsiella pneumoniae and Klebsiella oxytoca among hospitalized patients. *Period biol.* 2016;118(1):53–8. 10.18054/pb.2016.118.1.3160

[16] Ejaz H Ul-Haq I Mahmood S : Detection of extended-spectrum beta-lactamases in Klebsiella pneumoniae: comparison of phenotypic characterization methods. *Pak J Med Sci.* 2013;29(3):768–72. 10.12669/pjms.293.3576 24353625 PMC3809290

[17] Pavez M Troncoso C Osses I : High prevalence of CTX-M-1 group in ESBL-producing enterobacteriaceae infection in intensive care units in southern Chile. *Braz J Infect Dis.* 2019;23(2):102–10. 10.1016/j.bjid.2019.03.002 31028724 PMC9425662

[18] Quan J Zhao D Liu L : High prevalence of ESBL-producing Escherichia coli and Klebsiella pneumoniae in community-onset bloodstream infections in China. *J Antimicrob Chemother.* 2017;72(1):273–80. 10.1093/jac/dkw372 27624571

[19] Sonda T Kumburu H Zwetselaar M van : Prevalence and risk factors for CTX-M gram-negative bacteria in hospitalized patients at a tertiary care hospital in Kilimanjaro, Tanzania. *Eur J Clin Microbiol Infect Dis.* 2018;37(5):897–906. 10.1007/s10096-018-3196-8 29464424 PMC5917002

