# Supplementary material for: Molecular Epidemiological Surveillance of CTX-M-15-producing Klebsiella pneumoniae from the patients of a teaching hospital in Sindh, Pakistan
Source: F1000Res. 2021 Nov 15;10:444. Originally published 2021 Jun 4. [Version 3] doi: 10.12688/f1000research.53221.3 (PMC8596178; doi:10.12688/f1000research.53221.3)
Supplement: Peer review history

# Peer review: Reviewer response for version 3

**DOI:** 10.5256/f1000research.79331.r100184

**Published:** 2021-11-16

**Authors:** Mubashir Zafar

**Affiliations:** 1 Aga Khan University, Karachi, Pakistan

All comments are addressed. I recommend article for indexing.

Is the work clearly and accurately presented and does it cite the current literature?

Partly

If applicable, is the statistical analysis and its interpretation appropriate?

No

Are all the source data underlying the results available to ensure full reproducibility?

Partly

Is the study design appropriate and is the work technically sound?

No

Are the conclusions drawn adequately supported by the results?

No

Are sufficient details of methods and analysis provided to allow replication by others?

No

Reviewer Expertise:

NA

I confirm that I have read this submission and believe that I have an appropriate level of expertise to confirm that it is of an acceptable scientific standard.

# Peer review: Reviewer response for version 3

**DOI:** 10.5256/f1000research.79331.r100186

**Published:** 2021-11-16

**Authors:** Abbas Farahani

**Affiliations:** 1 Infectious and Tropical Diseases Research Center, Hormozgan Health Institute, Hormozgan University of Medical Sciences, Bandar Abbas, Iran

All comments and corrections have been considered by the authors *.*

Is the work clearly and accurately presented and does it cite the current literature?

No

If applicable, is the statistical analysis and its interpretation appropriate?

No

Are all the source data underlying the results available to ensure full reproducibility?

No

Is the study design appropriate and is the work technically sound?

No

Are the conclusions drawn adequately supported by the results?

Yes

Are sufficient details of methods and analysis provided to allow replication by others?

No

Reviewer Expertise:

Microbiology

I confirm that I have read this submission and believe that I have an appropriate level of expertise to confirm that it is of an acceptable scientific standard.

# Peer review: Reviewer response for version 2

**DOI:** 10.5256/f1000research.58553.r92304

**Published:** 2021-08-27

**Authors:** Mubashir Zafar

**Affiliations:** 1 Aga Khan University, Karachi, Pakistan

The article is good but it needs major revision.

 First, the title of the article should be revised as Epidemiological Surveillance of CTX-M-15-producing *Klebsiella pneumoniae* among patients in teaching hospital (add city and country name).

**Major revision**

**Abstract**

- The objective is different from the title of the study.
- Don’t use the abbreviation in the abstract.
- The method in the abstract is incomplete.
- The result and conclusion are also incomplete information in the abstract.

**In the introduction section**

- Relevant studies are absent, the rationale of the study is very weak

**In method section**

- Study setting, sampling technique, sample size were absent.
- The data collection procedure is incomplete and not standard.
- Data analysis should be revised.

**In result section**

- A demographic table is not needed and should be deleted.

**The discussion section is incomplete**

- Limitation of study is absent
- Conclusion of study should be revised
- Reference should be the latest 

**Minor revision**

- The article would benefit from being revised for formatting and use of language. 

Is the work clearly and accurately presented and does it cite the current literature?

Partly

If applicable, is the statistical analysis and its interpretation appropriate?

No

Are all the source data underlying the results available to ensure full reproducibility?

Partly

Is the study design appropriate and is the work technically sound?

No

Are the conclusions drawn adequately supported by the results?

No

Are sufficient details of methods and analysis provided to allow replication by others?

No

Reviewer Expertise:

Preventive Medicine specialist

I confirm that I have read this submission and believe that I have an appropriate level of expertise to state that I do not consider it to be of an acceptable scientific standard, for reasons outlined above.

# Peer review: Reviewer response for version 2

**DOI:** 10.5256/f1000research.58553.r90226

**Published:** 2021-08-02

**Authors:** SUPRIT DESHPANDE

**Affiliations:** 1 HIV Vaccine Translational Research Laboratory, NCR Biotech Science Cluster, Translational Health Science & Technology Institute, Faridabad, Haryana, India

There is no mention about ESBL positive *K. pneumoniae *in the Figure 1A description/legend, whereas the same has been described in the running text.

 unaddressed

 Clinical relevance of demographic characteristics with the results obtained in this study is not described clearly by the authors in discussion section. This needs to be elaborated."

 unaddressed

 As per the study it’s not only Carbapenems but also quinolones (Ciprofloxacin) also that showed their effectiveness with 100% sensitivity. Authors has missed to point it out and describe about it in the running text.

 unaddressed

 Relevant references needs to be quoted for conventional bacteriological techniques in methods

 unaddressed

 As per the Fig 1E, the authors have tested different classes of antibiotics, here what was the rationale behind choosing these specific antibiotics? The results illustrates that the more percent resistance was mainly observed in the antibiotics belongs to class of cephalosporins, is there any clinical relevance for this kind of observation, this needs to be discussed in the discussion section with relevant references if available.

 unaddressed

 Authors mentioned in the method that the genomic DNA was separated upon boiling the bacterial colony at 98 degrees Celsius and the same DNA was used for PCR amplification of ESBL genes. The ESBL genes are mainly encoded by plasmid DNA. Here, in this study the authors used which DNA (bacterial genomic DNA or plasmid DNA) for PCR amplification to understand the genetic diversity of ESBL genes

 Needs to be addressed

 The highest percent of ESBL was found in blood specimens, is there any scientific reason for this? If yes, it needs to be described in the discussion part.

 Unaddressed

 Authors mentioned in the method that the genomic DNA was separated upon boiling the bacterial colony at 98 degrees Celsius and the same DNA was used for PCR amplification of ESBL genes. The ESBL genes are mainly encoded by plasmid DNA. Here, in this study the authors used which DNA (bacterial genomic DNA or plasmid DNA) for PCR amplification to understand the genetic diversity of ESBL genes?

 Unaddressed

Is the work clearly and accurately presented and does it cite the current literature?

Yes

If applicable, is the statistical analysis and its interpretation appropriate?

I cannot comment. A qualified statistician is required.

Are all the source data underlying the results available to ensure full reproducibility?

Partly

Is the study design appropriate and is the work technically sound?

Yes

Are the conclusions drawn adequately supported by the results?

Yes

Are sufficient details of methods and analysis provided to allow replication by others?

Partly

Reviewer Expertise:

HIV molecular virology , immunology and antibody discovery

I confirm that I have read this submission and believe that I have an appropriate level of expertise to confirm that it is of an acceptable scientific standard.

# Peer review: Reviewer response for version 2

**DOI:** 10.5256/f1000research.58553.r90227

**Published:** 2021-07-28

**Authors:** Abbas Farahani

**Affiliations:** 1 Infectious and Tropical Diseases Research Center, Hormozgan Health Institute, Hormozgan University of Medical Sciences, Bandar Abbas, Iran

The revised manuscript has been improved, however, before it can be accepted for publication in the journal, I require of authors to revise it before indexing. Comments to authors:

- Please provide the accession numbers for ESBL genotypes.
- In general, the discussion can be structured better. There is a substantial overlap and repetition between the Result and the Discussion sections.

Is the work clearly and accurately presented and does it cite the current literature?

No

If applicable, is the statistical analysis and its interpretation appropriate?

No

Are all the source data underlying the results available to ensure full reproducibility?

No

Is the study design appropriate and is the work technically sound?

No

Are the conclusions drawn adequately supported by the results?

Yes

Are sufficient details of methods and analysis provided to allow replication by others?

No

Reviewer Expertise:

Microbiology

I confirm that I have read this submission and believe that I have an appropriate level of expertise to confirm that it is of an acceptable scientific standard, however I have significant reservations, as outlined above.

# Peer review: Reviewer response for version 1

**DOI:** 10.5256/f1000research.56580.r88330

**Published:** 2021-07-05

**Authors:** Abbas Farahani

**Affiliations:** 1 Infectious and Tropical Diseases Research Center, Hormozgan Health Institute, Hormozgan University of Medical Sciences, Bandar Abbas, Iran

**Summary:**

 This research article seeks to isolate and characterize ESBL-producing *K. pneumoniae* from inpatients and outpatients. The ESBL genotype and molecular relatedness of strains identified here were done using standard and acceptable methods and procedures. *K. pneumoniae* isolates were identified in 35.18% (n=513) of all samples. More than half (69.9%) of all isolates were ESBL producers and all of the ESBL-positive *K. pneumoniae *isolates had one or more ESBL genes. Their study showed that CTX-M-15 was the major prevalent ESBL type among the isolates.

**Comments: **

- Teaching hospital needs description of the hospital, number of beds, number of admission and other information about the hospital is required.
- The authors claimed that to have used the CLSI guideline, while reference number 8 is related to the European Committee on Antimicrobial Susceptibility Testing (EUCAST)?
- Where did you source your antibiotics from?
- The concentration of each reaction in PCR must be added.
- Please provide information about quantifying and qualify DNA samples.
- Please add company and country for SPSS software.
- The sequenced products of the isolates need to be deposited in a database (i.e. Genbank) and accession numbers need to be given.
- Provide the accession numbers of the sequenced products in the article.
- In Table 2; mean ± SD is 47 ± 17.5 while in text is 48.5 ± 8.9 years? Please explain. 
- In the title of the article the authors have mentioned that isolates collected from inpatients and outpatients of a teaching hospital, but in the main text is no mention of them and the number of isolates in each group is not mentioned? Please explain.
- Finally, I recommend merging both Results and Discussion sections for better clarity and impact, and move to Rapid Communication submission.

Is the work clearly and accurately presented and does it cite the current literature?

No

If applicable, is the statistical analysis and its interpretation appropriate?

No

Are all the source data underlying the results available to ensure full reproducibility?

No

Is the study design appropriate and is the work technically sound?

No

Are the conclusions drawn adequately supported by the results?

Yes

Are sufficient details of methods and analysis provided to allow replication by others?

No

Reviewer Expertise:

Microbiology

I confirm that I have read this submission and believe that I have an appropriate level of expertise to state that I do not consider it to be of an acceptable scientific standard, for reasons outlined above.

# Response

**Published:** 2021-07-08

**Authors:** Saeed Sattar Shaikh

**Affiliations:** Imam Abdulrahman Bin Faisal University, Saudi Arabia

We thank the reviewer for taking time and reviewing our article. We have revised our manuscript to address the comments. Please, find below our responses to reviewer comments.

- *Teaching hospital needs description of the hospital, number of beds, number of admission and other information about the hospital is required.*
- **Author’s Response:** Details of hospital updated.
- *The authors claimed that to have used the CLSI guideline, while reference number 8 is related to the European Committee on Antimicrobial Susceptibility Testing (EUCAST)?*
- **Author’s Response:** Replaced with new reference.
- *Where did you source your antibiotics from?*
- **Author’s Response:** Updated the details of antibiotics.
- *The concentration of each reaction in PCR must be added.*
- **Author’s Response:** PCR reaction mixture details are added.
- *Please provide information about quantifying and qualify DNA samples.*
- **Author’s Response:** The information and data were not mentioned because, for PCR reaction purified DNA was not required and crude DNA was sufficient for detection.
- *Please add company and country for SPSS software.*
- **Author’s Response:** Updated the details of SPSS software.
- *The sequenced products of the isolates need to be deposited in a database (i.e. Genbank) and accession numbers need to be given.*
- **Author’s Response:** As per our Institutional policy, we have to deposit all research data to our institution. Hence it takes much administrative procedures before the institution deposits it to GenBank.
- *Provide the accession numbers of the sequenced products in the article.*
- **Author’s Response:** As per our Institutional policy, we have to deposit all research data to our institution. Hence it takes much administrative procedures before the institution deposits it to GenBank.
- *In Table 2; mean ± SD is 47 ± 17.5 while in text is 48.5 ± 8.9 years? Please explain.*
- **Author’s Response:** The correct one is 48.5 ± 8.9 years; and is now corrected.
- *In the title of the article the authors have mentioned that isolates collected from inpatients and outpatients of a teaching hospital, but in the main text is no mention of them and the number of isolates in each group is not mentioned? Please explain.*
- **Author’s Response:** Authors trying to indicate; Inpatient and outpatients as all patients attending the hospitals. We have updated the title of the article to avoid confusion.
- *Finally, I recommend merging both Results and Discussion sections for better clarity and impact and move to Rapid Communication submission.*
- **Author’s Response:** We do not have any problem in merging results and discussion if journal format allows us.

# Peer review: Reviewer response for version 1

**DOI:** 10.5256/f1000research.56580.r86850

**Published:** 2021-07-05

**Authors:** SUPRIT DESHPANDE

**Affiliations:** 1 HIV Vaccine Translational Research Laboratory, NCR Biotech Science Cluster, Translational Health Science & Technology Institute, Faridabad, Haryana, India

In this manuscript “Characterization of CTX-M-15-Klebsiella pneumoniae from inpatients and outpatients of a teaching hospital” by Muzaheed *et al.*, the authors studied and surveyed the prevalence of ESBL producing genes in *Klebsiella pneumoniae* infections. The authors have screened *K. pneumoniae* from 1458 clinical samples from the individuals enrolled under a different study. Increase in number of cases due to bacterial drug resistance has become a severe cause of global concern. Here, authors have screened the *K. pneumoniae *isolates and with the help of genotyping they tried to identify the distribution of different class of ESBL genes and their prevalence.

1. This paper imparts more of a kind “molecular epidemiological surveillance” of ESBL producing genes in one of the predominant nosocomial pathogen K. *pneumoniae*, whereas the title of the paper is about characterization of one of the ESBL producing genes (CTX-M). Author needs to re-think about the title of this paper as the existing title seems not suitable for the kind of study actually done and reported through this manuscript
2. Since the samples were not specifically collected for this study, then the inclusion and exclusion criteria would have been not as per this current study? This needs to be clarified. Also, it would be better to shift the entire sentence about sample collection from isolation and identification paragraph to the paragraph on participants inclusion and exclusion criteria.
3. Under methods, the second paragraph on sample collection, inclusion and exclusion criteria needs to be shifted first and the present first paragraph on isolation and identification needs to be shifted second.
4. Double parentheses for ESR in inclusion and exclusion criteria paragraph in methods section need to be removed.
5. Author mentioned Kirby bar method, it needs to be mentioned as Kirby Bar Disk diffusion method.
6. Under methods section in the paragraph on ESBL screening and confirmation by phenotypic methods, in the last but one line the standard disc diffusion where “method” is missing needs to be written.
7. There is no mention about ESBL positive *K. pneumoniae *in the Figure 1A description/legend, whereas the same has been described in the running text.
8. Figure 1B is not described in the running text. Also in figure there is confusion with the numbering corresponds to the genes, for instance in TEM, SHV and CTX-M there should be three numbers (one number for each gene) but only two numbers are given along with the percentage. Please check and confirm.
9. Clinical relevance of demographic characteristics with the results obtained in this study is not described clearly by the authors in discussion section. This needs to be elaborated.
10. As per the study it’s not only Carbapenems but also quinolones (Ciprofloxacin) also that showed their effectiveness with 100% sensitivity. Authors has missed to point it out and describe about it in the running text.
11. The highest percent of ESBL was found in blood specimens, is there any scientific reason for this? If yes, it needs to be described in the discussion part.
12. What is the denominator for 411 males and 102 females clinical specimens from whom *K. pneumoniae* were isolated? If the sample sizes for male and female donors were different then before coming to the conclusion saying that in males it was higher than in females it needs to be proved statistically. Authors needs to clarify this.
13. As per the Fig 1E, the authors have tested different classes of antibiotics, here what was the rationale behind choosing these specific antibiotics?  The results illustrates that the more percent resistance was mainly observed in the antibiotics belongs to class of cephalosporins, is there any clinical relevance for this kind of observation, this needs to be discussed in the discussion section with relevant references if available.
14. Authors mentioned in the method that the genomic DNA was separated upon boiling the bacterial colony at 98 degrees Celsius and the same DNA was used for PCR amplification of ESBL genes. The ESBL genes are mainly encoded by plasmid DNA. Here, in this study the authors used which DNA (bacterial genomic DNA or plasmid DNA) for PCR amplification to understand the genetic diversity of ESBL genes?
15. Authors mentioned the amplicons were sequenced to identify the genetic diversity of different ESBL producing genes, I am not sure about the journal’s policy and whether these need to be submitted to the GenBank?
16. Relevant references needs to be quoted for conventional bacteriological techniques in methods.

Is the work clearly and accurately presented and does it cite the current literature?

Yes

If applicable, is the statistical analysis and its interpretation appropriate?

I cannot comment. A qualified statistician is required.

Are all the source data underlying the results available to ensure full reproducibility?

Partly

Is the study design appropriate and is the work technically sound?

Yes

Are the conclusions drawn adequately supported by the results?

Yes

Are sufficient details of methods and analysis provided to allow replication by others?

Partly

Reviewer Expertise:

HIV molecular virology , immunology and antibody discovery

I confirm that I have read this submission and believe that I have an appropriate level of expertise to confirm that it is of an acceptable scientific standard, however I have significant reservations, as outlined above.

# Response

**Published:** 2021-07-08

**Authors:** Saeed Sattar Shaikh

**Affiliations:** Imam Abdulrahman Bin Faisal University, Saudi Arabia

We thank you for your valuable comments and suggestions.  We have revised our manuscript accordingly. Please, find below our responses to reviewer comments.

- *This paper imparts more of a kind “molecular epidemiological surveillance” of ESBL producing genes in one of the predominant nosocomial pathogen K. pneumoniae, whereas the title of the paper is about characterization of one of the ESBL producing genes (CTX-M). Author needs to re-think about the title of this paper as the existing title seems not suitable for the kind of study actually done and reported through this manuscript*
- **Author’s Response:** Thank you for your suggestions we have modified title as suggested.
- *Since the samples were not specifically collected for this study, then the inclusion and exclusion criteria would have been not as per this current study? This needs to be clarified. Also, it would be better to shift the entire sentence about sample collection from isolation and identification paragraph to the paragraph on participants inclusion and exclusion criteria.*
- **Author’s Response:** The inclusion and exclusion criteria were set based on our focused organism that is *Klebsiella pneumoniae*. Also, we have shifted the entire sentence about sample collection from isolation and identification paragraph to the paragraph on participants inclusion and exclusion criteria.
- *Under methods, the second paragraph on sample collection, inclusion and exclusion criteria needs to be shifted first and the present first paragraph on isolation and identification needs to be shifted second.*
- **Author’s Response:** Updated shifted as suggested.
- *Double parentheses for ESR in inclusion and exclusion criteria paragraph in methods section need to be removed.*
- **Author’s Response:** Thank you, removed.
- *Author mentioned Kirby bar method, it needs to be mentioned as Kirby Bar Disk diffusion method.*
- **Author’s Response:** Thank you, updated as suggested.
- *Under methods section in the paragraph on ESBL screening and confirmation by phenotypic methods, in the last but one line the standard disc diffusion where “method” is missing needs to be written.*
- **Author’s Response:** Thank you, updated as suggested.
- *There is no mention about ESBL positive K. pneumoniae in the Figure 1A description/legend, whereas the same has been described in the running text.*
- **Author’s Response:** Thank you, updated figure legend as suggested.
- *Figure 1B is not described in the running text. Also in figure there is confusion with the numbering corresponds to the genes, for instance in TEM, SHV and CTX-M there should be three numbers (one number for each gene) but only two numbers are given along with the percentage. Please check and confirm.*
- **Author’s Response:** figure 1B is now mentioned in running text. The names of the genes have been written as per the standard procedures followed in previous literatures.
- *Clinical relevance of demographic characteristics with the results obtained in this study is not described clearly by the authors in discussion section. This needs to be elaborated.*
- **Author’s Response:** The aim and objectives of the study were to perform molecular epidemiology of the isolates regardless of their clinical sources and clinical presentations. This was beyond the scope of clinical correlations. However, existing data were being used for further study and analysis where additional publication is being tried.
- *As per the study it’s not only Carbapenems but also quinolones (Ciprofloxacin) also that showed their effectiveness with 100% sensitivity. Authors has missed to point it out and describe about it in the running text.*
- **Author’s Response:** Carbapenems are often the final influential therapy of choice to treat infections resulting from MDR Enterobacteriaceae that is why the authors have only concentrated on it.
- *The highest percent of ESBL was found in blood specimens, is there any scientific reason for this? If yes, it needs to be described in the discussion part.*
- **Author’s Response:** Because the clinical samples were mostly from blood.
- *What is the denominator for 411 males and 102 females clinical specimens from whom K. pneumoniae were isolated? If the sample sizes for male and female donors were different then before coming to the conclusion saying that in males it was higher than in females it needs to be proved statistically. Authors needs to clarify this.*
- **Author’s Response:** The dominator is 513, which were positive K. pneumoniae Out of which 411 were males and 102 females.
- *As per the Fig 1E, the authors have tested different classes of antibiotics, here what was the rationale behind choosing these specific antibiotics?  The results illustrates that the more percent resistance was mainly observed in the antibiotics belongs to class of cephalosporins, is there any clinical relevance for this kind of observation, this needs to be discussed in the discussion section with relevant references if available.*
- **Author’s Response:** The antibiotics were selected according to CLSI guidelines, Physician’s desk reference, Medical Letters and Peer-reviewed literature.
- *Authors mentioned in the method that the genomic DNA was separated upon boiling the bacterial colony at 98 degrees Celsius and the same DNA was used for PCR amplification of ESBL genes. The ESBL genes are mainly encoded by plasmid DNA. Here, in this study the authors used which DNA (bacterial genomic DNA or plasmid DNA) for PCR amplification to understand the genetic diversity of ESBL genes?*
- **Author’s Response:** Bacterial genomic DNA was used for PCR amplification. Whereas Trans-conjugation experiments were performed to identify conjugal resistance transfer. Which will be reported in next possible publication.
- *Authors mentioned the amplicons were sequenced to identify the genetic diversity of different ESBL producing genes, I am not sure about the journal’s policy and whether these need to be submitted to the GenBank?*
- **Author’s Response:** As per our Institutional policy, we have to deposit all research data to our institution. Hence it takes much administrative procedures before the institution deposits it to GenBank.
- *Relevant references needs to be quoted for conventional bacteriological techniques in methods.*
- **Author’s Response:** Thank you, reference added as suggested.
